# Looking at the recent advances in understanding α-synuclein and its aggregation through the proteoform prism

**DOI:** 10.12688/f1000research.10536.1

**Published:** 2017-04-20

**Authors:** Vladimir N. Uversky

**Affiliations:** 1 Department of Molecular Medicine and USF Health Byrd Alzheimerʼs Research Institute, Morsani College of Medicine, University of South Florida, 12901 Bruce B. Downs Blvd. MDC07, Tampa, FL, 33620, USA; 2 Laboratory of New Methods in Biology, Institute for Biological Instrumentation, Russian Academy of Sciences, 7 Institutskaya St., 142290 Pushchino, Moscow Region, Russian Federation; 3 Laboratory of Structural Dynamics, Stability and Folding Of Proteins, Institute of Cytology, Russian Academy of Sciences, 4 Tikhoretsky Av., 194064 St. Petersburg, Russian Federation

**Keywords:** α-synuclein, aggregation, synucleinopathies, multifunctionality

## Abstract

Despite attracting the close attention of multiple researchers for the past 25 years, α-synuclein continues to be an enigma, hiding sacred truth related to its structure, function, and dysfunction, concealing mechanisms of its pathological spread within the affected brain during disease progression, and, above all, covering up the molecular mechanisms of its multipathogenicity, i.e. the ability to be associated with the pathogenesis of various diseases. The goal of this article is to present the most recent advances in understanding of this protein and its aggregation and to show that the remarkable structural, functional, and dysfunctional multifaceted nature of α-synuclein can be understood using the proteoform concept.

## Introduction

**Figure 1.**
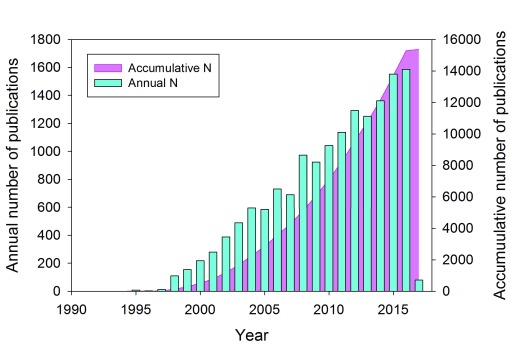
A time course of the development of interest in α-synuclein-related research. Web of Science data related to the publications dedicated to α-synuclein: the cumulative number of publications for the past 26 years (pink area plot) and the annual number of publications dedicated to this protein (cyan bars).

α-synuclein is a small, highly abundant, and highly conserved presynaptic protein with intimate links to many neurodegenerative diseases and is one of the more intensively studied human proteins. In fact, since its discovery in 1991 ^1^, followed by the demonstration of its natively unfolded nature ^2^, and especially after finding in 1997 a potential relationship between α-synuclein aggregation and the pathology of Parkinson’s disease (PD) ^3, 4^, this protein has attracted the close attention of many researchers specializing in various scientific areas. According to the Web of Science database, as of 17 February 2017, there were 15,367 publications about this protein, reflecting collective efforts of 37,066 researchers affiliated with 6,326 organizations from 93 countries/territories, and more than 3,140 papers were published on α-synuclein during the past 2 years alone. Figure 1 represents a more detailed view of these Web of Science data by showing the cumulative number of publications for the past 26 years and the annual number of publications dedicated to this protein.

Despite immense efforts, this 25-year-old enigmatic protein continues to keep multiple secrets related to its structure, function, dysfunction, multipathogenicity, and pathology transmission. The aforementioned considerations constitute a perfect stage for an important question related to α-synuclein: what is so special about this protein that makes it a multipathogenicity carrier? The goal of this article is to shed some light on α-synuclein-related mysteries by presenting the most recent advances in our understanding of this protein and its aggregation.

## Multipathogenicity angle

To better understand why researchers continue to study this protein, let’s consider what multipathogenicity means for α-synuclein. Although originally considered a potential cause of PD (where aggregated α-synuclein is present in the form of intracellular inclusions, Lewy bodies [LBs] and Lewy neurites [LNs] ^5, 6^), this protein is known to be involved in the pathogenesis of a diverse group of neurodegenerative diseases collectively known as synucleinopathies. Some of these maladies include Alzheimer’s disease (AD), neurodegeneration with brain iron accumulation type 1, pure autonomic failure, Down’s syndrome, amyotrophic lateral sclerosis-parkinsonism-dementia complex of Guam, multiple system atrophy (MSA), and several LB disorders (that, in fact, might represent a clinical continuum ^7^), such as sporadic and familial PD, dementia with LBs (DLB), diffuse LB disease, the LB variant of AD, and PD dementia ^4, 8– 15^. These (and potentially many other) neurodegenerative diseases can be considered α-synuclein-related brain amyloidoses, since all of them are characterized by the presence of common pathological intracellular inclusions containing α-synuclein in selectively vulnerable neurons and glia and since the onset and progression of their clinical symptoms, as well as the degeneration of affected brain regions, are linked to the formation of abnormal filamentous aggregates containing α-synuclein ^9, 15– 21^. Importantly, accumulated evidence indicates that α-synuclein-based aggregation and deposition of LBs can affect multiple areas of the peripheral and central nervous systems, such as the dorsal raphe nucleus, dorsal nucleus of the vagus nerve, hypothalamic nuclei, intermediolateral nucleus, locus coeruleus, nucleus basalis of Meynert, and substantia nigra ^22^, whereas LNs can be found in the basal ganglia, cerebral cortex, dorsal nucleus of the vagus nerve, and sympathetic ganglia as well as in the intramural autonomic ganglia of the gastrointestinal tract ^23, 24^. In line with these earlier observations, a recent study of the inclusion pathologies in PD and DLB confirmed the abundant presence of LBs and LNs in the dorsal motor vagal and solitary nuclei, locus coeruleus, parabrachial nuclei, pedunculopontine and raphe nuclei, periaqueductal gray, prepositus hypoglossal, substantia nigra, reticular formation, and ventral tegmental area and demonstrated the presence of LN in brainstem fiber tracts and the existence of LBs and LNs in cranial nerve, premotor oculomotor, precerebellar, and vestibular brainstem nuclei ^25^. This study also supported an important notion that α-synuclein deposition-related pathological processes can spread and do so transneuronally along anatomical pathways ^25^. Recently, application of the α-synuclein proximity ligation assay revealed the presence in the post-mortem brain tissue from PD patients of previously unrecognized pathology in the form of extensive diffuse deposition of α-synuclein oligomers that were often localized, in the absence of LBs, to neuroanatomical regions mildly affected in PD ^26^.

Obviously, although the diversity of synucleinopathies and related symptoms potentially can be attributed to the complexity of the organization of brain and nervous system (different motor and non-motor symptoms are the manifestation of the malfunction of different brain and nervous system regions), this complexity, in general, cannot be used to explain the cause of the multifaceted, α-synuclein-related pathology at the molecular level. In fact, since α-synuclein is highly expressed throughout the brain, accounting for as much as 1% of the total protein in soluble cytosolic brain fractions ^27^, and since any given synucleinopathy possesses unique spatiotemporal characteristics (happens at a specific time due to the malfunction of a specific brain region), some other factors, likely related to α-synuclein and its interaction with the environment (such as mutations in the *SYNC* gene, presence of alternatively spliced isoforms, post-translational modifications (PTMs), toxic insult, oxidative stress, presence of dopamine, metal ions, specific binding partners, etc.), should be taken into account.

## Connection of synucleinopathies to other diseases angle

Recent studies provided further support for an interesting molecular link between the synucleinopathies and other neurodegenerative diseases by demonstrating the ability of α-synuclein to interact with and regulate proteins specific to several degenerative maladies. For example, it was shown that α-synuclein can interact and co-aggregate with mutant huntingtin, a protein related to Huntington's disease ^28^, and with tau protein, the aggregation of which is associated with various tauopathies, including AD ^29^. A cooperation of α-synuclein with β-amyloid (Aβ) was shown to block SNARE-dependent vesicle fusion ^30^, whereas interaction with Aβ led to the inhibition of Aβ deposition and reduced plaque formation ^31^. Finally, α-synuclein was also shown to engage in interaction with autophagy/beclin1 regulator 1, a protein related to MSA pathogenesis, and this binding was dramatically enhanced by the α-synuclein phosphorylation at serine 129 ^32^. Note that the aforementioned interactions of α-synuclein with the proteins associated with other neurodegenerative diseases can be grouped into two classes – interactions leading to the co-aggregation of α-synuclein with said proteins and interactions leading to the modulation of functionality of proteins targeted by α-synuclein.

## Multifunctionality angle

Many papers in the field start with an introductory sentence stating that α-synuclein is a small, highly conserved presynaptic protein with unknown function. This statement is a bit odd, taking into account all the efforts of numerous researchers working on α-synuclein. In fact, according to the PubMed database (as of 16 December 2016), there were more than 7,150 papers mentioning synuclein function, many of those papers were dedicated to the detailed investigation of what this protein can do, and, as a result, many potential functions were ascribed to α-synuclein. The explanation of this contradiction (many functions are described, but function is unknown) is in the logistics of the classical structural biology relying on the influential “one gene – one enzyme – one function” hypothesis, according to which each gene encodes a single protein that has a unique biological function and is responsible for a single step in a metabolic pathway ^33^. In line with this hypothesis, distortion of normal protein function (in the form of loss of a normal function or gain of a pathological function) might represent the molecular basis of a proteinopathy.

However, since α-synuclein was shown to have not one but many functions (see below), this immediately brought significant uneasiness to functional data interpretation, leading to the logical conclusion that the observed multifunctionality indicates the lack of a unique function and, therefore, could be unreal. Although, from the viewpoint of the “one protein – one function” model, these observations provide grounds for the “protein with unknown function” epithet, an alternative (and much more intriguing and lucrative) hypothesis stating that α-synuclein is indeed a multifunctional protein, which can, in fact, do everything ascribed to it (and, probably, much more than that). In this case, problems with different α-synuclein-based functions might be directly or indirectly related to the pathogenesis of different synucleinopathies. In other words, a spectrum of functions can cause a spectrum of dysfunctions that might lead to a spectrum of diseases.

Early work in this direction showed that functions of α-synuclein can range from fatty acid binding ^34^ to interaction with plasma membranes and formation of membrane channels or modification of their activity ^35^, association with mitochondria causing mitochondrial dysfunction ^35^, metal binding ^36– 39^, interaction with pesticides and herbicides ^40– 42^, synaptic vesicle release and trafficking ^34^, positive and negative regulation of neurotransmitter release ^43^, regulation of certain enzymes and transporters ^34^, control of neuronal survival ^34^, regulation of the neuronal apoptotic response ^44^ and protection of neurons from various apoptotic stimuli ^44^, and promiscuous interaction with hundreds of unrelated proteins and other binding partners ^34, 45– 47^. In the past 2 years, the phenomenon of α-synuclein multifunctionality was further confirmed and elaborated by adding a long list of new functions. Note that the inventory below is far from being exhaustive, since over 1,100 articles dedicated to synuclein function were published during the last 2 years, and, therefore, it is physically impossible to cover all new developments in this commentary owing to the space restrictions.

### Knockout studies of the synuclein functionality

Knockout experiments, where the target gene is made inoperative, represent a useful approach for the evaluation of the global biological role of a query protein. Earlier studies showed that although α-synuclein-null mice were viable, fertile, and characterized by intact brain architecture, they possessed altered dopamine release and displayed a reduction in striatal dopamine and attenuated dopamine-dependent locomotor response ^48^. Furthermore, α-synuclein-null mice were characterized by a selective deficiency of undocked vesicles without affecting docked vesicles in hippocampal synapses ^49^, showed abnormal compartmentalization of norepinephrine in dentate gyrus ^50^, possessed greatly increased rates of operant behavior during intracranial self-stimulation ^51^, and showed an earlier onset of symptoms of experimental autoimmune encephalomyelitis ^52^ but were resistant to the Parkinsonian neurotoxin MPTP that inhibits mitochondrial complex I ^53^. This MPTP resistance was also present in the γ-synuclein and double α-synuclein/γ-synuclein-null mutant animals ^54^, and α-synuclein-null mice were shown to have an attenuated loss of striatal dopamine caused by prolonged chronic MPTP administration ^55^.

Multiple studies of the α-synuclein-knockout models suggested that this protein plays a role in lipid metabolism. In fact, α-synuclein-null animals were characterized by decreased brain palmitate uptake and altered palmitate metabolism ^56^, showed increased incorporation and turnover of the docosahexaenoic acid in brain phospholipids ^57^, possessed reduced arachidonate turnover in brain phospholipids ^58^, and showed increased mass of brain neutral lipids ^59^.

### Binding promiscuity

Among biological activities recently ascribed to α-synuclein is a numerous set of examples providing solid support for its reputation as a promiscuous binder. In fact, multiple new examples were reported showing that this protein is engaged in interaction with the multifunctional co-chaperone Bcl-2-associated athanogene-1 ^60^, prolyl oligopeptidase leading to enhanced α-synuclein dimerization ^61^, various synaptosomal proteins ^62^, mitochondria-associated membranes ^63^, molecular chaperone Munc18-1, which is a key component of the exocytic machinery that controls the release of neurotransmitters ^64^, the neuronal phosphoprotein synapsin III ^65^, voltage-dependent anion channel ^66^, a suicide inhibitor of monoamine oxidases, rasagiline ^67^, and the aldehyde of serotonin, 5-hydroxyindoleacetaldehyde ^68^.

### Control of cellular processes

Furthermore, α-synuclein has been linked to several cellular processes, such as activation of microglia ^69^, involvement in the regulation of autophagy ^70, 71^, initiation of innate and adaptive immune responses ^72^, membrane remodeling ^73, 74^, regulation of synaptic vesicle size ^75^, contribution to axonal transport impairment ^76^, stress-induced mitochondrial morphological remodeling (in cooperation with parkin and PINK1) ^77^, alteration of endoplasmic reticulum–mitochondrial communication ^78^, promotion of microtubule nucleation and enhancement of microtubule growth rate via interaction with microtubules and tubulin α _2_β _2_ tetramer ^79^, and inhibition of signaling related to the ATF6 pathway, which is a protective branch of the unfolded protein response ^80^, and even serving as a brain antimicrobial peptide that exhibits noticeable antibacterial activity against *Escherichia coli* and *Staphylococcus aureus* ^81^.

### α-synuclein radicals

Recently, Kumar *et al*. exploited an intriguing possibility that the formation of proteinaceous radicals might contribute to PD pathogenesis ^82^. Using the highly sensitive immuno-spin trapping technique, the authors showed that in the midbrains of maneb- and paraquat-co-exposed mice, the activation of NADPH oxidase and inducible nitric oxide synthase took place, eventually leading to the peroxynitrite-mediated formation of α-synuclein radicals in the dopaminergic neurons of exposed mice ^82^. Furthermore, the process of α-synuclein radical formation paralleled death dopaminergic neurons in the midbrains of maneb- and paraquat-co-exposed mice, indicating that there is an intricate link between protein radicals and disease progression ^82^.

### Regulation of various proteins

α-synuclein was also shown to regulate complexin-1 and midbrain-specific factor forkhead box P1 expression ^83^, enhance histone H3 lysine-9 (H3K9) dimethylation and promote H3K9me2-dependent transcriptional responses ^84^, protect the functions of Hsp90 clients, such as Akt and mTOR, when the activity of Hsp90 is blocked ^85^, stimulate protein phosphatase-2A (PP2A) activity and regulate tyrosine hydroxylase phosphorylation via the control of PP2A methylation ^86^, modulate the trafficking and function of glutamate N-methyl-d-aspartate receptor ^87^, and promote Notch1 intracellular domain degradation via interaction with the ubiquitin E3 ligase Fbw7 ^88^.

### α-synuclein in the family circle

Synucleins constitute a family of closely related presynaptic proteins encoded by three distinct genes and are found only in vertebrates ^89^. In addition to α-synuclein, which is also known as the non-amyloid-β component (NAC) precursor protein or synelfin ^90– 92^, this family includes β-synuclein (phosphoneuroprotein 14 or PNP14) ^92– 94^, and γ-synuclein (breast cancer-specific gene 1 [ *BCSG1*] or persyn) ^95– 98^. There is 78% identity between human α- and β-synucleins, with the major difference between the two being the lack of the significant part of the NAC region ^13, 99^. Human γ-synuclein shares 60% sequence similarity with α-synuclein and lacks the tyrosine-rich C-terminal tail that represents a signature of α- and β-synucleins ^13, 99^. Importantly, in addition to the traditional α-synuclein-containing pathological inclusions, the development of several synucleinopathies is accompanied by the appearance of α-, β-, and γ-synuclein-positive vesicular-like lesions at the presynaptic axon terminals in the hippocampal dentate, hilar, and CA2/3 regions ^100^.

Structurally, all members of the synuclein family are intrinsically disordered at physiological conditions but adopt comparable partially folded conformations at acidic pH or at high temperature ^101^. Both α- and γ-synucleins can easily form fibrils under a variety of identical conditions ^101^, whereas β-synuclein fibrillation requires the presence of some metal ions (Zn ^2+^, Pb ^2+^, and Cu ^2+^) ^102^.

Fibrillation of human α-synuclein can be inhibited by the addition of either β- or γ-synuclein *in vitro* ^101^, with β-synuclein being shown to inhibit α-synuclein aggregation in an animal model ^103^. NMR paramagnetic relaxation enhancement experiments revealed that β-synuclein forms transient heterodimers with α-synuclein that are characterized by high specificity and weak affinity ^104^. Recently, the competition of β-synuclein for binding sites at the surfaces of lipid vesicles and at the surfaces of α-synuclein fibrils was proposed as a molecular mechanism of the β-synuclein-driven inhibition of lipid-induced α-synuclein aggregation and secondary nucleation of α-synuclein fibrillation, respectively ^105^. In agreement with this inhibitory action of β-synuclein, some positive results of the use of β-synuclein as a means for reducing aggregated α-synuclein levels were obtained in preclinical studies ^106^.

Double-knockout of α- and β-synucleins in mice did not impair basic brain functions or survival, and no significant changes were detected in the ultrastructure of synuclein-deficient synapses, in synaptic plasticity, or in the pool size of synaptic vesicles ^107^. However, these double null animals were characterized by the decreased dopamine levels in their brains and showed selective changes in the levels of synaptic signaling proteins, such as complexins and 14-3-3 proteins ^107^. Recently, analysis of the triple α-synuclein/β-synuclein/γ-synuclein-knockout mice revealed that these proteins have profound effects on the presynaptic architecture and serve as “important orchestrators of presynaptic terminal topography”, regulating presynapse size and organization of the pool of synaptic vesicles ^108^. A systematic analysis of the structural and functional features of the nigrostriatal system in mice with every possible combination of knockout members of the synuclein family revealed that although these proteins have noticeable functional redundancy, some functions are specific for a particular member and therefore the remaining synucleins cannot fully compensate for the deficiency of a lost family member ^109^. For example, β-synuclein was shown to be needed for the efficient maintenance of balance and coordination in aged animals, whereas the presence of α-synuclein is crucial for stabilization of the striatal dopamine level and cannot be compensated by other family members ^109^.

## Regulatability angle

Also, a set of recent studies was dedicated to the analysis of various means of regulation of α-synuclein, its normal and pathological functions, and aggregation. It was shown that this protein can be regulated, at the expression level, by the microRNAs miR-34b and miR-34c ^110^ as well as miR-19b, miR-29a, and miR-29c ^111^, by promoter methylation in the α-synuclein gene *SNCA* ^112^, and by other means of epigenetic-mediated regulation ^113^. Aggregation of α-synuclein can be controlled by antibodies at substoichiometric concentrations (as low as 1:1000 antibody to protein ratio) ^114^. Different monoclonal anti-C-terminal antibodies were shown to differently interact with different forms of α-synuclein (monomeric or aggregated), with the antibodies with the strongest binding to aggregated protein also being the strongest inhibitors of α-synuclein fibrillation and membrane permeabilization ^115^. Aggregation of α-synuclein can be modulated via interaction with the moonlighting 14-3-3 proteins that act as ATP-independent anti-aggregation “holdases” ^116^, by caspase-1-mediated truncation ^117^, by the small secretory chaperone proSAAS ^118^, or by *Geum urbanum* extract ^119^. The aggregation, toxicity, levels, and secretion of α-synuclein were shown to be controlled by endocytic recycling pathway components, such as Rab8b, Rab11a, Rab13, and Slp5 ^120^. Also, interaction with an anti-amyloidogenic agent, ginsenoside Rg1, was shown to inhibit the fibrillation and toxicity of α-synuclein and disaggregate preformed fibrils ^121^. The extracellular α-synuclein can be sorted in extracellular vesicles in a SUMOylation-dependent manner ^122^, whereas the extracellular release of this protein is controlled by the DnaJ/Hsc70 chaperone complexes ^123^. The nuclear accumulation and toxicity of α-synuclein can be regulated by a novel protein, TRIM28 ^124^, that controls the fate specification of the neural cells ^125^, whereas loss of lysosomal β-glucocerebrosidase activity promotes global intracellular accumulation and toxicity of α-synuclein ^126^.

## Structural polymorphism angle

### Protein-chameleon

The immense multifunctionality and related multipathogenicity of α-synuclein suggest that this protein should have a highly amenable structure to be able to do everything ascribed to it. In agreement with this hypothesis, it was recognized early on that α-synuclein is a typical intrinsically disordered protein (IDP) that does not have a stably folded structure under physiological conditions ^2, 127^. Furthermore, α-synuclein can serve as an illustrative example of the protein-chameleon concept ^128^, where protein has a highly pliable structure that is extremely sensitive to the environmental conditions. Such a protein is able to morph under the action of numerous factors. As a result, α-synuclein may "stay substantially unfolded, or adopt an amyloidogenic partially folded conformation, or fold into α-helical or β-structural species, both monomeric and oligomeric. Furthermore, it might form several morphologically different types of aggregates, including oligomers (spheres or doughnuts), amorphous aggregates, and or amyloid-like fibrils" ^128^.

The intrinsically disordered nature of α-synuclein is not only a property of the purified protein *in vitro* but also preserved *in vivo*, as shown by the analysis of this protein endogenously expressed in the central nervous system, erythrocytes, and mammalian cell lines using native and denaturing gel electrophoresis techniques, size-exclusion chromatography, and an oligomer-specific ELISA ^129^. Compelling support for this idea was also provided by the results of the in-cell NMR analysis of human protein expressed in bacteria ^130^. Very recently, further evidence of the preservation of the disordered nature of monomeric α-synuclein under physiological cell conditions was given by the application of the in-cell NMR and electron paramagnetic resonance spectroscopy to derive the atomic-level information on the structure and dynamics of α-synuclein in different mammalian non-neuronal and neuronal cells ^131^. Based on the molecular dynamics studies, it has also been concluded that a disordered monomer represents the dominant state within the structural ensemble of α-synuclein ^132^.

### Residual structure

It was pointed out that although α-synuclein behaves as a highly disordered protein, the solution conformation of this protein is not a random coil but contains some residual structure ^127, 133^. This residual structure is extremely sensitive to various environmental factors ^134^. For example, based on the in-cell NMR analysis, it was recently concluded that the conformations of α-synuclein in the cellular environment are more compact than those of the isolated protein *in vitro* ^131^. These more compact *in vivo* conformations provide efficient shielding of the residues of the aggregation-prone NAC region, thereby counteracting spontaneous aggregation ^131^ and supporting the “functional misfolding” hypothesis, according to which a polypeptide chain of an IDP can spontaneously misfold “to sequester the preformed elements inside the non-interactive or less-interactive cage, therefore preventing these elements from the unnecessary and unwanted interactions with non-native binding partners” ^135^.

### Effects of post-translational modifications

Similar to many other IDPs, α-synuclein is subject to numerous PTMs, such as phosphorylation, ubiquitination, SUMOylation, O-GlcNAcylation, N-terminal acetylation, nitrosylation, and truncation, among which phosphorylation, truncation, and ubiquitination are believed to be the major disease-associated PTMs ^136, 137^. Since α-synuclein might have multiple PTMs affecting different residues, and since multiple sites can be subjected to a given PTM (e.g. phosphorylation might occur at S87, S129, or Y125, whereas K12, K21, or K23 can be subjected to ubiquitination), this protein is likely to be highly heterogeneous in its native state. This PTM-driven heterogeneity creates an important problem related to understanding the role of individual PTMs in the structural properties, normal function, misfolding, aggregation, and dysfunction of α-synuclein. Besides the identification of the enzymes involved in regulating these PTMs, a solution to this problem requires the preparation of site-specifically modified proteins. A breakthrough in this direction was achieved in Lashuel’s group, who elaborated a set of enzymatic, synthetic, and semisynthetic strategies for the site-specific and tightly controlled introduction of PTMs at single or multiple sites ^138– 142^. These approaches pave the way for generating pure and homogenously modified samples of α-synuclein.

### Effects of familial PD mutations

Among the important factors responsible for the modulation of α-synuclein’s residual structure are familial PD mutations. In addition to “traditional” PD mutations (such as A53T ^3^, A30P ^143^, and E46K ^144^) that have been known to the scientific community for a relatively long time, several “new” disease-associated mutations in α-synuclein (H50Q ^145, 146^, G51D ^147, 148^, and A53E ^149^) were discovered over the past 3 years. These mutations were shown to differently modulate α-synuclein functions and aggregation propensity. For example, the formation of non-fibrillar aggregate (such as oligomers or protofibrils) and not fibrils was accelerated by the A30P mutation ^150, 151^. Two other “traditional” PD mutants, A53T and E46K, were both shown to be characterized by accelerated fibrillation ^150, 152– 154^. Similarly, aggregation and fibrillation of the H50Q mutant were dramatically accelerated ^155^. On the other hand, significant reduction in α-synuclein oligomerization and fibrillation was induced by the G51D and A53E mutations, with the G51D mutant shown to form amorphous aggregates ^148, 156^, and with the A53E mutant being able to eventually form very thin amyloid fibrils ^156– 158^.

It is known that although mutations can modulate the aggregation propensity of α-synuclein, the global structure of this IDP is rather insensitive to mutations ^150, 153, 157, 159– 163^. Furthermore, based on the results of the extensive molecular dynamics simulations in aqueous solution, it has been concluded that fibril structure was not affected by PD-related mutations (A30P, E46K, H50Q, G51D, A53E, and A53T), but the relative stabilities of fibrillar structures and their conformational preferences were altered by mutations ^164^. However, the dynamics and residual structure present in the conformational ensembles of monomeric α-synuclein were clearly affected by the familial PD mutations ^150, 153, 157, 159– 163^. For example, by strategic placing of tryptophan residues, the effect of three PD mutations (A53T, E46K, and A30P) on the site-specific structural dynamics of this protein was analyzed ^165^. This analysis showed that mutations affected local conformational flexibility, microenvironment, and solvent exposure ^165^. Analysis of the effects of various mutations on the dynamics of the α-synuclein conformational ensemble revealed that the intramolecular diffusion of this protein is differently affected by aggregation-promoting and aggregation-inhibiting mutations, being correspondingly either drastically slowed down or accelerated in comparison with that of the wild-type protein ^166^. To address the role of tyrosine residues on early stages of α-synuclein aggregation and the effects of these residues on protein dynamics, three modeled Tyr mutants (Y39A, Y133A, and Y125A/Y133A/Y136A) were subjected to all-atom molecular dynamics simulation ^167^. This analysis revealed that the residue Tyr133 plays an important role in driving intramolecular interactions between the hydrophobic residues in the N- and C-terminal regions and is crucial for the fibrillation process ^167^.

## Polymorphism of aggregated forms of α-synuclein angle

Not only is monomeric α-synuclein characterized by structural polymorphism but also its oligomeric and insoluble aggregated forms are polymorphic at both structural and functional levels. For example, application of the cryo-electron microscopy image reconstruction technique revealed that oligomers that are kinetically trapped during α-synuclein fibril formation are characterized by different sizes, β-sheet contents, and levels of exposed hydrophobicity ^168^. Also, two morphologically different oligomeric α-synuclein forms were found in human post-mortem PD brain tissue ^169^. The presence of two different types of oligomeric species formed by the wild-type α-synuclein and its familial PD-related mutants A30P, E46K, and A53T was described using hydrogen/deuterium exchange monitored by mass spectrometry ^170^. Although generated at specific conditions, oligomers of the wild-type α-synuclein and its familial PD-related mutants A30P, A53T, E46K, H50Q, and G51D have similar global structures and are composed of a similar number of monomers, but they interact differently with biological membranes ^171^.

Several recent observations are in line with the idea that oligomeric/fibrillar forms of α-synuclein might be functionally different, indicating the presence of a functional polymorphism among the aggregated polymorphs. For example, it was found that fibrillar α-synuclein can trigger inflammatory responses, whereas oligomeric forms of this protein were unable to initiate these cascades ^172^. Although both oligomeric and fibrillar forms of α-synuclein are able to generate free radicals, only the oligomeric protein can reduce endogenous glutathione and cause subsequent neuronal toxicity ^173^. Also, microtubule assembly was shown to be efficiently inhibited by fibrillar α-synuclein, and this fibril-mediated inhibitory effect was greater than that of the oligomeric species (protofibrils) ^174^.

Slight changes in the environmental conditions during the fibrillation process were shown to cause the formation of morphologically different amyloid fibrils with distinctive molecular characteristics that can be inherited by the next generation of fibrils through self-propagation ^175^. Under specific conditions, pure forms of structurally different fibrillar polymorphs of α-synuclein were obtained and characterized structurally and functionally ^176^. In addition to different morphologies, these polymorphs were characterized by different structures, different levels of toxicity, different *in vivo* and *in vitro* seeding and propagation properties ^176^, different molecular arrangement in the unit cell, and distinct dynamic features ^177^. Subsequent structural and physical characterization of several fibrillar polymorphs assembled from α-synuclein supported the notion that the same protein can assemble into a variety of large particles with fibrillar shapes, that fibrillar polymorphs are morphologically and nanomechanically different, and that exceptional structural and physical polymorphism is present within the fibrillar form of this protein ^178^. These findings provide mechanical foundation for better understanding of the nature of different α-synuclein strains ^179^ that define the ability of the aggregated α-synuclein to be engaged in different neurodegenerative diseases ^176^.

## Strains and transmission angle

It is important to note that the phenomenon of polymorphism of amyloid fibrils is not an exclusive property of α-synuclein but was described for other amyloidogenic proteins ^180, 181^ and was linked to the phenomenon of strains originally found in mammalian prion protein ^182– 185^ and now described for α-synuclein ^179^, yeast and fungal prions ^186– 188^, transthyretin ^189^, and insulin ^190^ as well as Aβ ^191^ and tau protein ^192^. Strain phenomenon is related to the peculiarities of amyloid propagation and transmission, where different aggregated forms of a protein can cause the development of different pathologies. In relation to the subject of this article, the ability of α-synuclein to aggregate into distinct high-molecular-weight assemblies was proposed to be associated with the ability of this protein to cause different synucleinopathies ^179^. This idea is supported by the direct observation of the induction of different synucleinopathies after the administration of the different α-synuclein strains (oligomers, ribbons, and fibrils) by injection into rat brain ^193^.

Another analogy of aggregated α-synuclein to prion protein is its “infectivity”, i.e. the ability to be efficiently transmitted between neurons, thereby supporting the pathological spread within the affected brain during disease progression (e.g. as described by Braak's staging criteria of PD ^194, 195^). Although original models describing interneuronal propagation suggested some passive release of aggregated α-synuclein from injured, degenerated, or dead neurons, a recent study showed that intact, relatively healthy neurons are engaged in efficient cell-to-cell passage of α-synuclein ^196^. It was also indicated that endosomal processing might serve as a point of convergence between the transcellular propagation of aggregated α-synuclein and the intracellular trafficking of this protein ^197^. A crucial role of high-affinity binding of preformed α-synuclein fibrils to the lymphocyte-activation gene 3 protein is for the initiation of endocytosis and transmission of aggregated α-synuclein, thereby propagating its toxicity and leading to the loss of dopamine neurons and the development of biochemical and behavioral deficits *in vivo* ^198^. Importantly, it is not only exogenous fibrillar forms of α-synuclein that are able to promote cellular pathologies, since soluble amyloid oligomers that precede LB formation were also linked to the cell-to-cell transmission of α-synuclein pathology ^199^.

## Concluding remarks: proteoforms to the rescue

**Figure 2.**
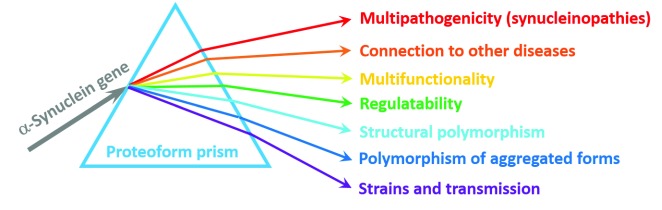
Proteoform concept as a prism for looking at and understanding α-synuclein. Schematic representation of the proteoform concept using the analogy of a prism. Here, an “incident light” (α-synuclein gene, *SNCA*), while going through the proteoform “prism” (a set of mechanisms put forward to generate different proteoforms), is “diffracted”, giving rise to a “spectrum” of proteoforms (a set of chemically or structurally different forms of a protein) for conducting different functions.

The knowledge of the peculiar behavior of α-synuclein accumulated over the past two decades provides an illustration of an important notion that the complexity of a biological system is mostly determined by its proteome size and not by the genome size ^200^. In fact, the number of functionally different proteins found in eukaryotic organisms is substantially higher than the number of genes. The functional diversification of proteinaceous products of a gene is achieved by several means, including the allelic variations (i.e. single or multiple point mutations, indels, SNPs) at the DNA level, alternative splicing, and other pre-translational mechanisms affecting mRNA, complemented by a wide spectrum of various PTMs of a polypeptide chain. As a result of all of these events, a set of distinct protein molecules can be created from a single gene, giving rise to the proteoform concept ^201^. Recently, it was proposed that in addition to the aforementioned means that increase the chemical variability of a polypeptide chain, the protein’s structural diversity can be further increased by some other mechanisms, such as intrinsic disorder and function ^202^. In this view, a correlation between protein structure and function is considered a “protein structure–function continuum”, where a given protein exists as a dynamic conformational ensemble containing multiple proteoforms (conformational/basic, inducible/modified, and functioning) characterized by diverse structural features and miscellaneous functions ^202^. In application to α-synuclein, the protein structure–function continuum idea is further enhanced by the presence of remarkable structural and functional variability of its self-aggregated forms. Therefore, due to its ability to oligomerize and aggregate, an intrinsically disordered α-synuclein is characterized by the enormously expanded set of proteoforms where each of the various monomeric, oligomeric, and insoluble species is further split into numerous conformational/basic, inducible/modified, and functioning proteoforms. In other words, as illustrated by Figure 2, consideration of α-synuclein through the proteoform prism provides a logical explanation for its remarkable structural, functional, and dysfunctional multifaceted nature.

Outlined in this article, the proteoform-centric view of α-synuclein represents an important shift in understanding the biology and pathology of this protein. In fact, despite the enormous amount of effort expended by the scientific community to find a single most important physiological function of α-synuclein, there is no clear understanding of the unique biological role of this protein as of yet. However, as it follows from a great deal of the published work on α-synuclein, the applicability of the “one sequence – one structure – one function” paradigm to this protein is questionable. In other words, it seems that the search for a single physiological function of α-synuclein may be entirely misplaced, since the intrinsically disordered nature of the protein, which defines the structural polymorphism of the conformational ensemble of its monomeric form, bestows multifunctionality upon α-synuclein. Logical consequences of this structural heterogeneity and multifunctionality of a monomeric protein are the structural polymorphism of aggregated states and a “spectrum of dysfunctions” that define a range of diseases. Therefore, the enormous structural and functional complexity of α-synuclein has serious implications for future studies to understand the biology of this protein and for the development of future therapeutic strategies targeting α-synuclein.

## Abbreviations

Aβ, β-amyloid; AD, Alzheimer’s disease; DLB, dementia with Lewy bodies; H3K9, histone H3 lysine-9; IDP, intrinsically disordered protein; LB, Lewy body; LN, Lewy neurite; MSA, multiple system atrophy; NAC, non-amyloid-β component; PD, Parkinson’s disease; PP2A, protein phosphatase-2A; PTM, post-translational modification.
